# Antimicrobial susceptibilities of *Neisseria gonorrhoeae* in Canada, 2020

**DOI:** 10.14745/ccdr.v48i1112a10

**Published:** 2022-11-03

**Authors:** Robyn Thorington, Pamela Sawatzky, Brigitte Lefebvre, Mathew Diggle, Linda Hoang, Samir Patel, Paul Van Caessele, Jessica Minion, Richard Garceau, Myrna Matheson, David Haldane, Genevieve Gravel, Michael R Mulvey, Irene Martin

**Affiliations:** 1 National Microbiology Laboratory Branch, Public Health Agency of Canada, Winnipeg, MB; 2 Laboratoire de santé publique du Québec, Ste-Anne-de- Bellevue, QC; 3 Provincial Laboratory of Public Health Alberta, Edmonton, Alberta, Canada; 4 British Columbia Centres for Disease Control Public Health Microbiology & Reference Laboratory, Vancouver, BC; 5 Public Health Ontario Laboratory, Toronto, ON; 6 Cadham Provincial Laboratory, Winnipeg, MB; 7 Roy Romanow Provincial Laboratory, Regina, SK; 8 Dr. Georges L. Dumont University Hospital Centre, Moncton, NB; 9 Government of the Northwest Territories, Yellowknife, NT; 10 Queen Elizabeth II Health Science Centre, Halifax, NS; 11 Surveillance and Epidemiology Division, Centre for Communicable Diseases and Infection Control Branch, Public Health Agency of Canada, Ottawa, ON

**Keywords:** gonorrhea, *Neisseria gonorrhoeae*, antimicrobial resistance, antimicrobial susceptibility, national surveillance system, passive surveillance

## Abstract

**Background:**

The Gonococcal Antimicrobial Surveillance Programme is a passive surveillance system that has monitored antimicrobial resistance in *Neisseria gonorrhoeae* in Canada since the 1980s. This article summarizes the demographics, antimicrobial resistances and NG-MAST (*N. gonorrhoeae* multiantigen sequence typing) for cultures collected in 2020.

**Methods:**

The National Microbiology Laboratory (NML) in Winnipeg received resistant *N. gonorrhoeae* cultures from provincial and territorial public health laboratories. Agar dilution was used to determine the minimum inhibitory concentrations to ten antimicrobials for all cultures received at NML, according to Clinical and Laboratory Standards Institute guidelines. The NG-MAST typing was also determined for each culture.

**Results:**

A total of 3,130 *N. gonorrhoeae* cases were cultured across Canada in 2020; a 36% decrease from 2019 (n=4,859). The level of decreased susceptibility to cefixime increased significantly between 2016 and 2020 to 2.8% (*p*=0.0054). Decreased susceptibility to ceftriaxone declined significantly between 2016 (1.8%) and 2020 to 0.9% (*p*=0.001), and there was no significant change with azithromycin between 2016 (7.2%) and 2020 (6.1%). The proportion of cultures with an azithromycin minimum inhibitory concentrations of ≥1 mg/L increased significantly from 11.6% in 2016 to 15.3% in 2020 (*p*=0.0017). The most common NG-MAST type in Canada for 2020 was sequence type (ST)-11461, while ST-12302 was most commonly associated with azithromycin resistance and ST-16639 with cephalosporin decreased susceptibility.

**Conclusion:**

Antimicrobial resistance in *N. gonorrhoeae* remains an important public health concern and continued surveillance is imperative to monitor trends to ensure the recommended therapies will be the most effective.

## Introduction

*Neisseria gonorrhoeae* is the causative agent of gonorrhoeae, which is the second most reported bacterial sexually transmitted infection (STI) in Canada. In 2019, there were 35,443 cases reported in Canada; more than double the number of cases reported in 2014 ((1)). Similarly, the incidence of infections has increased from 45.9/100,000 to 94.3/100,000 during that timeframe ((2)).

Due to the ability of *N. gonorrhoeae* to evolve and develop resistance to antimicrobials that are used to treat infections, the World Health Organization released a global action plan to control the spread and impact of antimicrobial resistance (AMR) *N. gonorrhoeae* in 2012 and the Canadian Antimicrobial Resistance Surveillance System advised caution with regards to multidrug-resistant gonorrhea in 2020 ((3–5)). Of particular concern are isolates with either decreased susceptibility to third-generation cephalosporins or resistance to azithromycin, which are part of the currently recommended treatment regimen of ceftriaxone (250 mg intramuscularly plus azithromycin 1 g orally) ((6)). In Canada, there were two cases of cephalosporin-resistant *N. gonorrhoeae* between 2017 and 2020 and several cases of high-level azithromycin resistance ((1,7,8)).

Since the 1980s, the Gonococcal Antimicrobial Surveillance Programme has run as a passive national surveillance program. Isolates that are submitted to this program undergo antimicrobial susceptibility testing and are characterized using *N. gonorrhoeae* multiantigen sequence typing (NG-MAST). The NG-MAST uses highly variable regions of the *porB* gene (PIB porin) and the *tbpB* gene (subunit B of transferrin-binding protein) alleles for molecular epidemiology of *N. gonorrhoeae*. The NG-MAST is a molecular typing method and can be used in outbreak investigations and to support treatment failure investigations. It has also shown a close association between a subset of sequence types (STs) and antimicrobial resistance, including azithromycin resistance and ST-12302 in Canada ((9–11)).

Gonorrhea most often presents as urethritis in males and cervicitis in females, though females are more likely to be asymptomatic ((12)). If cases of gonorrhoea are untreated, the bacterium can enter the blood and other sterile sites causing disseminated gonococcal infections (DGI). While uncommon, DGI cases can have severe morbidity, causing arthritis, dermatitis, migratory polyarthralgia, tenosynovitis and, in rare cases, endocarditis ((13,14)).

Antimicrobial resistant *N. gonorrhoeae* is continually evolving and new resistances can rapidly emerge. Continued surveillance of antimicrobial susceptibility and STs of *N. gonorrhoeae* is necessary to identify clusters, inform treatment guidelines and mitigate the impact of resistant gonorrhea. The severe acute respiratory syndrome coronavirus 2 (SARS-CoV-2) pandemic, which was declared by the World Health Organization in early 2020, decreased the testing capacity of laboratories across Canada for *N. gonorrhoeae* culture; the number of isolates analyzed compared to previous years greatly decreased. This article summarizes the antimicrobial susceptibility trends and sequence typing of *N. gonorrhoeae* cultures in Canada for 2016–2020.

## Materials and methods

### Surveillance

Surveillance of *N. gonorrhoeae* AMR in Canada consists of a voluntary passive laboratory system where provincial and territorial partners send *N. gonorrhoeae* cultures to the National Microbiology Laboratory (NML). Isolates cultured between January 1 and December 31, 2020, were received from Alberta, British Columbia, Manitoba, New Brunswick, Northwest Territories, Nova Scotia, Ontario, Québec and Saskatchewan. In 2020, a total of 3,130 *N. gonorrhoeae* isolates were cultured in Canada: 1,628 viable cultures that were resistant to at least one antibiotic were submitted to NML for antimicrobial susceptibility testing and molecular typing; 1,089 cultures were tested by provincial and territorial laboratories and antimicrobial susceptibility testing results were submitted to NML. The remaining 413 presumed susceptible cultures that were tested by provincial and territorial laboratories in 2020 were not submitted to NML but were included in the final denominator used throughout this article. The total number of cultures from each province or territory and the number of cultures with resistance to at least one antimicrobial are given in **Table S1**. The main denominator used throughout this article is 3,130, unless otherwise noted.

### Isolate testing

All *N. gonorrhoeae* cultures received by NML (n=1,628) were tested for antimicrobial susceptibility using the agar dilution method to determine their minimum inhibitory concentrations (MICs) for ten antimicrobials (penicillin, tetracycline, erythromycin, spectinomycin, ciprofloxacin, ceftriaxone, cefixime, azithromycin, ertapenem and gentamicin). Interpretation of results are made in accordance with the Clinical and Laboratory Standards Institute, except for ceftriaxone and cefixime, which used the World Health Organization guidelines and erythromycin, ertapenem and gentamicin, which were based on publications ((4,15–19)). Penicillin, tetracycline, erythromycin and azithromycin were all resistant at a MIC ≥2 mg/L. Ciprofloxacin was resistant at a MIC of at ≥1 mg/L, gentamicin at a MIC of ≥32 mg/L, and spectinomycin at a MIC of ≥128 mg/L. Ceftriaxone has decreased susceptibility at a MIC ≥0.125 mg/L, cefixime has decreased susceptibility at a MIC of ≥0.25 mg/L, and ertapenem is non-susceptible at ≥0.063 mg/L (**Table S2**). Additional testing for the presence of β-lactamase was performed on all cultures received by NML and polymerase chain reaction detection of the *tetM* plasmid was done when tetracycline MICs were ≥16 mg/L. Isolates were categorized as susceptible, resistant, multidrug-resistant (MDR; either decreased susceptibility or resistance to one recommended therapy plus at least two other antibiotics) or extensively drug-resistant (XDR; decreased susceptibility/resistance two currently recommended therapies plus resistance to at least two other antibiotics).

Cultures were also analyzed for molecular genotyping using NG-MAST ((10)). Sanger sequencing of both strands were assembled using SeqMan Pro 15 (DNAStar, Madison, Wisconsin, United States). Sequences were submitted to the PubMLST *Neisseria* spp. database to determine STs. Due to the decommissioning of the previous NG-MAST website (http://www.ng-mast.net), which resulted in the deletion of several thousand previously identified STs, some of the STs in this article contain updated allelic profiles from previous years.

### Data analysis

Demographic information submitted with the *N. gonorrhoeae* isolates included age, sex, isolation site, province and date of collection. Multiple isolates collected from the same patient within four weeks and with the same NG-MAST ST were considered to be duplicates. Determination of the isolate to be deemed a duplicate was based on a hierarchy of isolation sites, with isolates taken from a sterile site being first priority for inclusion (and marked as DGI), a throat isolate being second priority, followed by rectum, then the urogenital tract. For each figure, the denominator used is included in the footnote(s). The AMR trends for azithromycin, cefixime and ceftriaxone were analysed at both the provincial or territorial level and at the national level, while the correlation of the most common NG-MAST STs with AMR is also examined. Statistical significance of trends was assessed using the Cochran Armitage test of trend, with a *p*-value of <0.05 considered significant.

## Results

### Isolates tested, demographics and isolation sites

**Figure 1:**
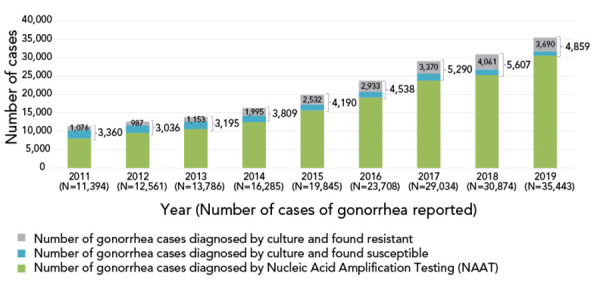
*Neisseria gonorrhoeae* cases in Canada, 2011 to 2019^a^ ^a^ Only 15%–20% of all gonorrhea cases were diagnosed by culture in Canada, the rest was detected using nucleic acid amplification test technology. Number of reported cases for 2020 had not yet been determined at the time of publication

Of the 3,130 isolates from across Canada in 2020, 70.1% had resistance to at least one antimicrobial (Table S1). In Canada over 80% of gonorrhoea cases were diagnosed using nucleic acid amplification tests (**Figure 1**), while the remaining ~20% cases were cultured ((20)). The technology for the prediction of antimicrobial susceptibility from a nucleic acid amplification test is complex and is currently offered as a laboratory-developed test by some research and reference laboratories, but the current gold standard requires culture.

In 2020, of those cultures sent to NML (n=2,679), 70.2% (n=1,880/2,679) came from individuals between the ages of 21 and 40 years, 21.9% (n=586/2,679) from individuals 41 years of age and older and 7.9% (n=213/2,679) from individuals younger than 21 years of age. The majority of isolates (82.9%; n=2,220/2,679), came from males, 16.6% (n=446/2,679) from females and 0.5% (n=13/2,679) came from either gender diverse or patients whose gender was not given. Most common overall isolation site for males was penis/urethra (60.9%, n=1,352/2,220) while for females it was the throat (32.1%, n=143/446). For more details on ages of patients and isolation sites see **Table S3**.

### Antimicrobial resistance trends in antimicrobials not included in the recommended treatment guidelines 2016–2020

**Figure 2:**
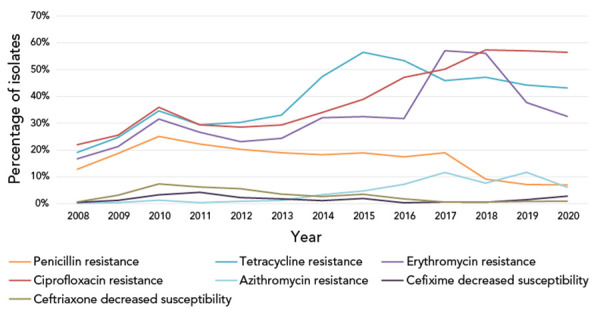
Percentage of antimicrobial resistance of *Neisseria gonorrhoeae* tested in Canada, 2008–2020^a,b^ ^a^ Percentages are based on the total number of isolates tested nationally: 2008=3,907; 2009=3,106; 2010=2,970; 2011=3,360; 2012=3,036; 2013=3,195; 2014=3,809; 2015=4,190; 2016=4,538; 2017=5,290; 2018=5,607; 2019=4,859; 2020=3,130 ^b^ Due to some provinces not testing all seven antimicrobials in 2017, 2018 and 2019 penicillin denominators were 3,267, 3,883, 3,822 and 2,409, respectively; erythromycin denominators were 2,879, 3,418, 3,446 and 2,025, respectively; and tetracycline denominator in 2020 was 2,409

National trends of gonococcal antimicrobial susceptibilities for 2008–2020 indicated that of the antimicrobials that were not currently part of the recommended treatment regimens, ciprofloxacin was the only one to have seen a continuing increase in the level of resistance in recent years, increasing from 22.0% in 2008 to 56.5% in 2020. Penicillin resistance peaked in 2010 at 25.1% but fell to 7.0% in 2020. Tetracycline resistance decreased from 56.4% in 2015 to 43.1% in 2020. Erythromycin resistance fell from its peak at 57.0% in 2017 to 32.5% in 2020 (**Figure 2**).

### Cefixime antimicrobial resistance in Canada, 2016–2020

**Figure 3:**
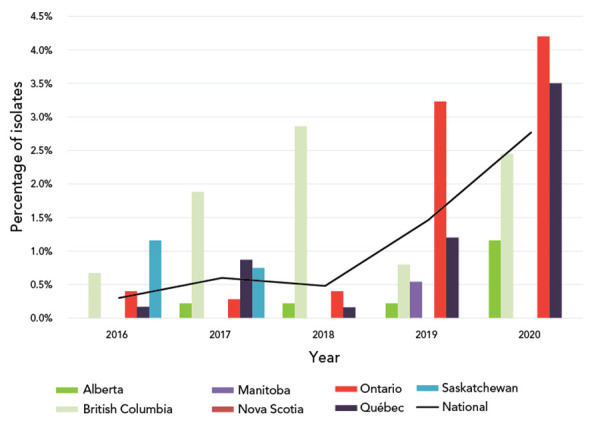
Percentage of *Neisseria gonorrhoeae* cultures with decreased susceptibility to cefixime by province, 2016–2020^a,b^ ^a^ Provinces included in this figure are only those that submitted at least one culture to the National Microbiology Laboratory that had decreased susceptibility to cefixime ^b^ Denominators used for the calculations of the percentages are the number of cultures tested in each province (data in Table S1)

Cefixime decreased susceptibility (CeDS, MIC ≥0.25 mg/L) has seen a significant increase (*p*=0.0054) from 0.30% in 2016 to 2.8% in 2020, which is almost double from 1.5% in 2019 (**Figure 3**). The proportion of strains with higher MICs (≥0.25 mg/L) increased significantly during this timeframe as well (*p*=0.0054), see **Table S4** for complete break down of the proportion of MICs. The MDR strains with CeDS also increase significantly (*p*<0.0001) (**Figure S1**).

### Ceftriaxone antimicrobial resistance in Canada, 2016–2020

**Figure 4:**
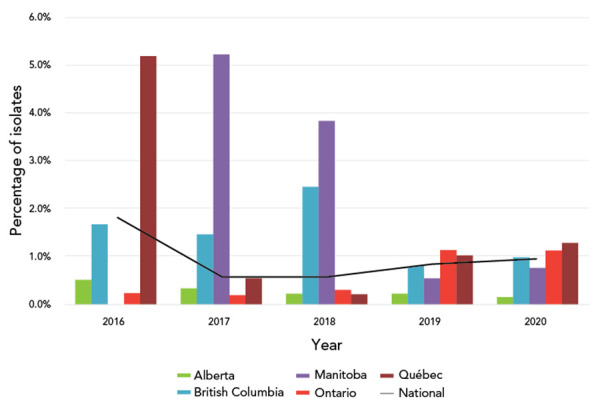
Percentage of *Neisseria gonorrhoeae* cultures with decreased susceptibility to ceftriaxone by province, 2016–2020^a,b^ ^a^ Provinces included in this figure are only those that submitted at least one culture to the National Microbiology Laboratory that had decreased susceptibility to ceftriaxone ^b^ Denominators used for the calculations of the percentages are the number of cultures tested in each province (Table S1)

Ceftriaxone decreased susceptibility (CxDS, MIC ≥0.125 mg/L) decreased significantly, falling from 1.8% in 2016 to 0.9% in 2020 (*p*=0.001) (**Figure 4**). The proportion of MDR isolates with CxDS decreased significantly (*p*<0.0001) as well, from 18.2% in 2016 to 4.6% in 2020. The proportion of MDR isolates with both CeDS and CxDS increased significantly (*p*<0.0001) (Figure S1) from 1.2% in 2016 to 10.0% in 2020.

### Azithromycin antimicrobial resistance in Canada, 2016–2020

**Figure 5:**
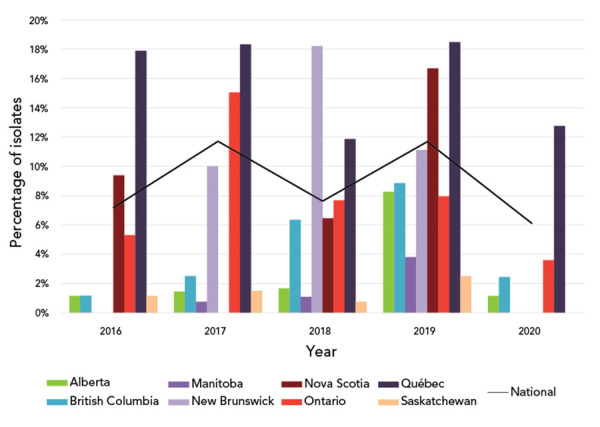
Percentage of azithromycin-resistant *Neisseria gonorrhoeae* cultures by province, 2016–2020^a,b^ ^a^ Provinces included in this figure are only those that submitted at least one culture to the National Microbiology Laboratory that was azithromycin resistant ^b^ Denominators used for the calculations of the percentages are the number of cultures tested in each province (Table S1). Newfoundland and Labrador had one azithromycin resistant isolate in 2019

**Figure 6:**
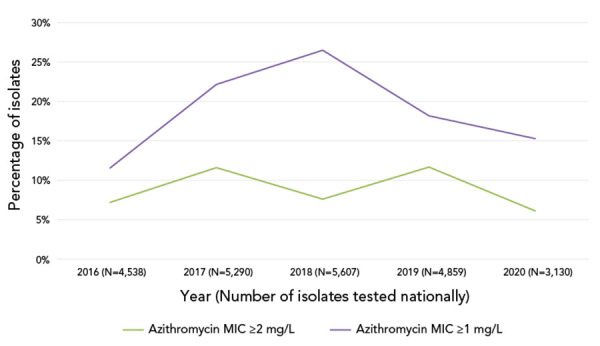
Trends the percentage of azithromycin minimum inhibitory concentrations for *Neisseria gonorrhoeae* at the susceptibility breakpoint^a^ Abbreviation: MIC, minimum inhibitory concentration ^a^ Denominators used for the calculations of the percentages are the number of cultures tested in each province (Table S1). Disseminated gonococcal infections cases in Canada, 2016–2020

Azithromycin resistance (AziR) did not change significantly from 2016 to 2020 for cultures that had a MIC ≥2 mg/L as shown in **Figure 5**. For cultures that had a MIC ≥1 mg/L, there was a significant increase (*p*=0.0017) from 11.6% in for 2016 to 15.3% in 2020 (**Figure 6**).

The number of MDR cultures that were AziR increased significantly from 78.3% in 2016 to 97.0% in 2020 (*p*<0.0001) (Figure S1). Between 2016 and 2020, there was a significant decrease in the number of MDR cultures (*p*=0.0117) from 8.9% to 6.3% (**Figure S2**). There was no significant change in the number of XDR cultures between 2016 (n=1) and 2020 (n=2) (**Figure S3**). A full list of all XDR cases found in Canada is given in **Table S5**.

Within Canada over the past five years there has been a significant increase (<0.0001) in DGI cases from 0.03% (n=6/23,708) in 2016 to 0.20% (n=71/35,443) in 2020.

### NG-MAST sequence type trends in Canada, 2016–2020

**Figure 7:**
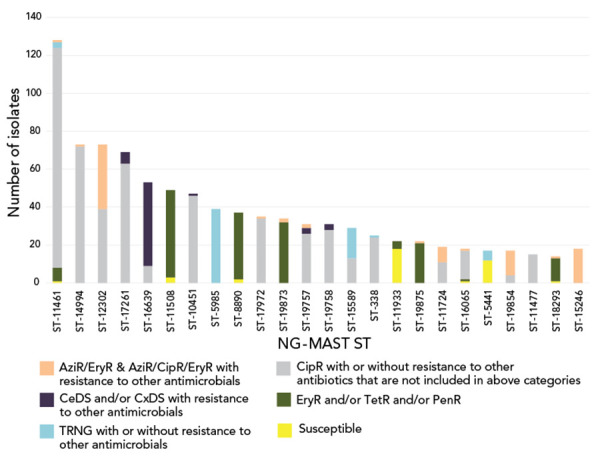
Distribution of resistance characterization within *Neisseria gonorrhoeae* NG-MAST sequence types, 2020, n=1,590^a^ Abbreviations: AziR, azithromycin resistance; CeDS, cefixime decreased susceptibility; CipR, ciprofloxacin resistance; CxDS, ceftriaxone decreased susceptibility; EryR, erythromycin resistance; NG-MAST, *N. gonorrhoeae* multiantigen sequence typing; ST, sequence type; PenR, penicillin resistant; TerR, tetracycline resistant; TRNG, tetracycline resistant *Neisseria gonorrhoeae* ^a^ Does not include nine isolates that were non-typeable. This graph represents 915 isolates. The remaining 674 isolates are dispersed among 279 sequence types containing one to 14 isolates each

In total, 1,590 cultures were successfully typed for NG-MAST in 2020. The most frequently detected NG-MAST sequence type in Canada was ST-11461 (n=128), followed by ST-14994 (n=73) and ST-12302 (n=73). As shown in **Figure 7**, ST-12302, ST-11724, ST-19854 and ST-15246 all have high proportions of the cultures that are AziR. The ST-16639 has a high proportion of cultures with either CeDS or CxDS. The number of isolates for each ST that were from each province and territory is shown in **Figure S4**, while **Figure S5** shows the trends of some common STs over time. Of note is the sharp decrease in the number of ST-12302 and ST-14994 in 2020 (Figure S5).

## Discussion

On March 11, 2020, the World Health Organization declared the outbreak of the coronavirus, SARS-CoV-2, a global pandemic ((21,22)). This global emergency had a cascading effect on all aspects of public health and infectious disease surveillance. From the laboratory perspective, due to the redistribution of laboratory personnel in response to the SARS-CoV-2 pandemic, STI laboratory testing numbers dropped dramatically across Canada, with multiple jurisdictions suspending their STI testing entirely at times throughout 2020 ((22,23)). This redistribution of labour led to a decrease in the total number of *N. gonorrhoeae* cultures collected in public health laboratories across Canada by 36% between 2019 and 2020; from 4,859 cultures in 2019 to 3,130 in 2020 (Table S1). While the number of reported gonorrhea cases in Canada in 2020 has not yet been reported, multiple countries have reported estimates on 1) the impact on surveillance of gonorrhea AMR, 2) adherence to recommended treatment guidelines and 3) under-reporting of cases of STIs in 2020 due to the lockdowns and reassignment of laboratory staff ((24–26)). The full effect of the SAR-CoV-2 pandemic on the surveillance of *N. gonorrhoeae* AMR will not be fully known for several years ((27)).

Decreased susceptibility to cefixime had been declining in Canada and Europe since the early 2010s ((28–30)). While more recent data from Europe has not yet been published, Canada has seen a rapid and significant increase in the level of gonococcal isolates with CeDS since 2018 (Figure 3). What is driving this increase is unclear, although there has been an increase in ST-16639, and the majority of these cultures have a cefixime MIC ≥0.25 mg/L. This ST was first detected in Canada in 2019 (n=38) and increased in 2020 (n=53). This trend in ST-16639 isolates should be carefully monitored going forward to inform public health actions.

Another factor that could be contributing to this increase in CeDS is a possible increase in the use of oral therapy, specifically using the combination therapy of 800 mg cefixime plus 1 g azithromycin during the various lockdowns that occurred across Canada in 2020. Because cefixime is an oral medication (versus the intramuscular injection delivery required for ceftriaxone) it is simpler for delivery to patients during times of limited health services and telehealth appointments.

The national level of AziR in Canada did not differ significantly between 2017 and 2020, although there was some variability from year to year. Part of this annual variability is due to geographical variability in AziR, with some regions having now updated their treatment protocols in response to these data ((31)). The effects of these updated treatment recommendation on the AziR rates in those regions will be determined with continued surveillance. Much of the increase in AziR levels between 2013 and 2018 was driven by ST-12302, which has a strong association with low-level resistance to azithromycin ((11)). Since 2017, the number of ST-12302 cultures sent to NML has decreased steadily, which could be a factor in the plateauing of AziR.

While the percentage of cultures with azithromycin MICs at or above the break point of 2 mg/L has remained steady since 2017, the number of *N. gonorrhoeae* cultures with a MIC of 1 mg/L has increased significantly during that time (Figure 6). The cause of this shift is unclear, though in NML’s whole genome sequencing data, when looking at the single-nucleotide polymorphisms that are associated with AziR, many strains contain the mosaic *mtrR* promoter, which is associated with decreased susceptibility to azithromycin in *N. gonorrhoeae* ((32)). While there is potentially an ongoing shift in azithromycin MIC in Canada, being driven by the prominence of the mosaic *mtrR* promoter, other jurisdictions, most notably Australia, have set their breakpoint for azithromycin at 1 mg/L, which is also the epidemiological cut-off value from European Committee on Antimicrobial Susceptibility Testing ((33,34)). While Canada has not seen an increase in reported treatment failures for gonorrhea, due to dual therapy being the recommended treatment method, this rise in azithromycin MIC 1 mg/L is of concern and should be monitored.

Since 2016, there has been a national increase in the number of DGI cases. While this increase is uneven across Canada, more emphasis on detection, investigation and culturing of these cases should be made. What differentiates an uncomplicated *N. gonorrhoeae* infection from one that becomes a DGI is still unclear, although there is some evidence that it is linked to certain *N. gonorrhoeae* virulence factors, particularly *porB* protein structures type “A”, due to its role in the interaction of the complement system ((35)). This can lead to the ability of *N. gonorrhoeae* to spread to sterile sites throughout the body, which can cause far greater morbidity. Provinces and territories across Canada should consider more closely monitoring and tracking these serious cases.

## Limitations

An important limitation to consider when interpreting the data presented in this article is that submission of isolates is voluntary and is not standardized across the country; therefore, the overall interpretation of the results is difficult due to the limitations related to the isolates available for testing. Only a subset of laboratory isolates from each province may have been submitted for testing; thus, this article does not reflect true incidence or rates of antimicrobial resistance in Canada.

Due to the SAR-CoV-2 pandemic and the reallocation of laboratory resources that followed, there was a decrease in the number of *N. gonorrhoeae* cultures that were grown across Canada and submitted to NML. This might have led to some trends being over- or under-reported due to the differing surveillance capabilities in each of the provinces and territories throughout the pandemic.

## Conclusion

Though the number of isolates collected decreased in 2020 in comparison to previous years, *N. gonorrhoeae* AMR remains an important public health concern. In the past five years, there has been a significant increase in the proportion of cultures with decreased susceptibility to cefixime, a significant increase in the number of DGI cases across the country, and a change in the most prevalent NG-MAST ST. Significant changes were not seen with antimicrobials. Continued surveillance of *N. gonorrhoeae* AMR in Canada is imperative to monitor these trends, as well as to detect clonal outbreaks, to identify new or emerging types of antimicrobial resistance and to help to ensure that national treatment guidelines will continue to advise effective treatment regimens. Enhancing surveillance to include linked epidemiological and laboratory data would address the limitations regarding data representativeness and interpretation in the current passive surveillance system. The Enhanced Surveillance of Antimicrobial Resistant Gonorrhea was initiated in 2014 and has been implemented to fill this gap.

## Supplemental material

These documents can be accessed on the Supplemental material file.
Table S1: Summary of the *Neisseria gonorrhoeae* cultures, submitted antimicrobial resistance testing results, and laboratory data received by the National Microbiology Laboratories from participating provinces and territory, 2016–2020
Table S2: *Neisseria gonorrhoeae* agar dilution antimicrobial testing ranges and minimum inhibitory concentration interpretations
Table S3: Age of patient and isolation site of the *Neisseria gonorrhoeae* cultures tested at the National Microbiology Laboratory, 2020 (n=2,679)
Table S4: Cefixime susceptibilities of *Neisseria gonorrhoeae* isolates tested by the National Microbiology Laboratory, 2016–2020
Figure S1: Percentage MDR-GC cultures in Canada between 2016 and 2020 broken down by whether they are resistant to azithromycin or if they have decreased susceptibility to either cefixime or ceftriaxone
Figure S2: Trends of multi-drug resistant *Neisseria gonorrhoeae* in Canada from 2016 to 2020
Figure S3: Trends of extensively drug-resistant *Neisseria gonorrhoeae* in Canada from 2016 to 2020
Table S5: All extensively drug-resistant *Neisseria gonorrhoeae* strains isolated in Canada (N=29)
Figure S4: Provincial distribution within *Neisseria gonorrhoeae* NG-MAST sequence types, 2020 (N=1,590)
Figure S5: Trends of prevalent NG-MAST sequence types of *Neisseria gonorrhoeae* isolates tested by the National Microbiology Laboratory, 2016–2020
